# Scavengers of reactive γ-ketoaldehydes extend *Caenorhabditis elegans* lifespan and healthspan through protein-level interactions with SIR-2.1 and ETS-7

**DOI:** 10.18632/aging.101011

**Published:** 2016-08-09

**Authors:** Thuy T. Nguyen, Samuel W. Caito, William E. Zackert, James D. West, Shijun Zhu, Michael Aschner, Joshua P. Fessel, L. Jackson Roberts

**Affiliations:** ^1^ Department of Pharmacology, Vanderbilt University, Nashville, TN 37232, USA; ^2^ Department of Molecular Pharmacology, Albert Einstein College of Medicine, Bronx, NY 10461, USA; ^3^ Department of Environmental Medicine, University of Rochester, Rochester, NY 14642, USA; ^4^ Division of Clinical Pharmacology, Department of Medicine, Vanderbilt University, Nashville, TN 37232, USA; ^5^ Division of Allergy, Pulmonary and Critical Care Medicine, Department of Medicine, Vanderbilt University Medical Center, Nashville, TN 37232, USA; ^6^ Department of Cancer Biology, Vanderbilt University, Nashville, TN 37232, USA

**Keywords:** *C. elegans*, aging, SIR-2.1, ETS transcription factors, oxidative stress

## Abstract

Isoketals (IsoKs) are highly reactive γ-ketoaldehyde products of lipid peroxidation that covalently adduct lysine side chains in proteins, impairing their function. Using *C. elegans* as a model organism, we sought to test the hypothesis that IsoKs contribute to molecular aging through adduction and inactivation of specific protein targets, and that this process can be abrogated using salicylamine (SA), a selective IsoK scavenger. Treatment with SA extends adult nematode longevity by nearly 56% and prevents multiple deleterious age-related biochemical and functional changes. Testing of a variety of molecular targets for SA's action revealed the sirtuin SIR-2.1 as the leading candidate. When SA was administered to a SIR-2.1 knockout strain, the effects on lifespan and healthspan extension were abolished. The SIR-2.1-dependent effects of SA were not mediated by large changes in gene expression programs or by significant changes in mitochondrial function. However, expression array analysis did show SA-dependent regulation of the transcription factor *ets-7* and associated genes. In *ets-7* knockout worms, SA's longevity effects were abolished, similar to sir-2.1 knockouts. However, SA dose-dependently increases *ets-7* mRNA levels in non-functional SIR-2.1 mutant, suggesting that both are necessary for SA's complete lifespan and healthspan extension.

## INTRODUCTION

Peroxidation of polyunsaturated fatty acids (PUFA) is a hallmark of oxidative stress, in part due to their susceptibility to free radical attack [1,2]. Accumulation of lipid peroxidation products has been implicated in the pathogenesis of a number of human diseases, such as atherosclerosis, cancer, and neurodegenerative diseases [3–5]. This phenomenon plays a critical role in the propagation of oxidative damage and in cell death cascades, in part through the formation of reactive aldehydes [6]. These secondary products of lipid peroxidation, which include malondialdehyde (MDA) and the reactive hydroxyl-alkenals, are known to contribute to and partially mediate the effects of lipid peroxidation [6,7].

Recent work at Vanderbilt has identified highly reactive levuglandin-like γ-ketoaldehydes (γ-KA, or isoketals, IsoK) comprised of 64 regio- and stereo-isomers. Isoketals are formed as products of the isoprostane pathway via rearrangement of prostaglandin H_2_-like endoperoxide intermediates (H_2_-isoprostanes) [8,9]. IsoKs covalently adduct ε-amino groups in lysyl residues of proteins to form stable adducts (structurally characterized as lactam rings) and intramolecular cross-links [9–11]. IsoK-lysyl-lactam adducts have been shown to be significantly increased in atherosclerosis, end-stage renal disease, Alzheimer's disease, and as a significant contributing cause of hypertension [12–14]. While the potent cytotoxicity of IsoKs and their ability to induce protein aggregation and to disrupt enzymatic function indicate a strong pathologic potential [15–18], meaningful investigation into the extent to which formation of IsoK adducts on proteins contributes to disease requires methods to selectively reduce the formation of IsoK adducts *in vivo*.

To better define the biological role of isoketals in oxidative injury and potentially prevent their detrimental effects, studies were performed to identify selective scavengers of IsoKs. A lead compound, pyridoxamine (PM) was identified through initial screens [19]. Structure-activity relationship studies identified the critical moiety to be a phenolic amine with the hydroxyl group adjacent to the methyl amine. Therefore, other phenolic amines such as salicylamine (SA) are similarly potent and as selective as PM for scavenging isoketals, but are more lipophilic. SA protects cellular viability in intact cells exposed to hydrogen peroxide, with SA pre-treatment leading to 5% occurrence in cell death, compared to 95% cell death in vehicle control treated cells, which suggests that IsoKs are major effectors of oxidative mediated death [20]. SA is orally bioavailable [21], and administering SA in mice prevents the age-related loss of working memory and the development of angiotensin II-induce hypertension [14,22]. Although preventing IsoKs from adducting to proteins has broad and remarkable beneficial biological effects, the precise molecular processes that are being altered by the IsoK scavengers are not clearly defined. More precisely, the potential role that IsoKs may play in the aging process, and how IsoK scavengers may be able to influence normal aging, is an open question currently.

Aging is characterized by progressively diminishing function at the molecular, cellular, tissue, and whole organism levels [23–26]. The overall results are a gradual decline in the capacity to respond to environmental challenges and an increasing vulnerability to disease and death [27–29]. The mechanisms contributing to the multi-level, multisystem changes that we recognize as aging are a matter of fairly intense debate. Broadly, mechanisms underlying aging can be divided into theories of programmed aging [30,31] and theories of cumulative damage [32] and failed homeostasis [33], though it must be recognized that the two may be related [34]. At least for the cumulative damage/failed homeostasis hypotheses (e.g., “rate of living”/metabolic theory [35,36], free radical theory [37], failed proteostasis [38], cumulative DNA damage [39], etc.), pathways controlling molecular metabolism and redox homeostasis repeatedly emerge as being central to the aging process [34]. One of the best studied pathways lying at the intersection of metabolic control, redox regulation, and aging is the sirtuin pathway.

Sirtuins are a highly conserved family of proteins that play major roles in adapting physiology to dietary extremes, as well as being implicated in countering aging and diseases associated with aging [40–42]. Sirtuins are nicotinamide adenine dinucleotide (NAD^+^)-dependent protein deacetylases and/or ADP-ribosyltransferases. Due to the requirement of NAD for biochemical activity, sirtuins sense and respond to the metabolic status of the cell. Indeed, this is thought to be a key mechanism by which caloric restriction extends natural lifespan – namely, by increasing NAD^+^ availability, which increases sirtuin activity. Numerous studies in model organisms, including yeast, worms, and flies, suggest that manipulation of sirtuin Sir2 (silent information regulator 2) and its homologs can extend lifespan [41,43–46]. Over-expression of the closest homolog to yeast Sir2 in *C. elegans*, *sir-2.1*, leads to extension of lifespan, and deletion or knockdown of the gene shortens lifespan [45,47–49]. Although it is a family of seven mammalian sirtuins (SIRT1-7) that play various roles in the regulation of stress resistance, metabolism, and cell survival, their roles in the regulation of mammalian lifespan are still unresolved. Despite the uncertainty, many studies suggest that sirtuins are a linchpin, regulating multiple homeostasis and stress response pathways – antioxidant defenses, energy metabolism, mitochondrial genomic maintenance, and others – that contribute to aging and age-related diseases.

In the present study, we tested the hypothesis that age-related oxidant injury and accumulation of IsoKs leads to adduction and inactivation of key proteins that regulate lifespan and healthspan. Specifically, we hypothesized that SIR-2.1 and downstream targets are inactivated by IsoK adduction, and that treatment with a scavenger of IsoKs would preserve protein function, extend natural lifespan and diminish degenerative changes in physical health. We found that treating wild-type Bristol N2 with SA, a potent scavenger of IsoKs, significantly extended lifespan and healthspan. We showed that SA's effects on longevity are dependent upon SIR-2.1, as knocking out its protein biochemical function abolished drug effect. In subsequent experiments, we have defined a SIR-2.1/*ets-7* axis that is preserved by SA to regulate lifespan and healthspan along with classical markers of aging and oxidant injury, largely through maintaining proteostasis.

## RESULTS

### Salicylamine increases lifespan and healthspan of wild-type adult *C. elegans*

**Figure 1:**
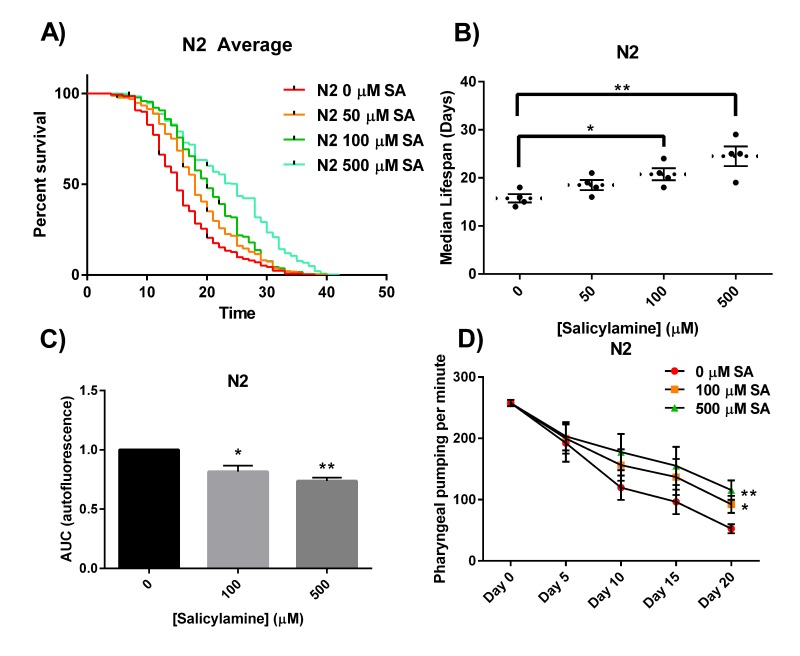
SA extends the lifespan of N2 *C. elegans* worms (**A**) Kaplan-Meier survival curves for concentration dependency of SA-mediated N2 lifespan extension. Upon day 1 of adulthood, SA was administered every 2 days and survival was assessed every other day until all the worms died. (**B**) Summary of SA treated N2 median lifespans. SA administration shows a dose-dependent increase in median lifespan. Data are expressed as means ± SEM from four independent experiments. *P < 0.05 as compared with vehicle control, **P < 0.01 as compared to vehicle control. (**C**) Effects of SA-mediated decreases in lipofuscin autofluorescence accumulation with age. SA response profiles were generated from integrating the area-under-the-curve (AUC) of fluorescent intensity as a function of time. Compared with N2 vehicle control, treatment with SA shows a significant reduction in autofluorescence. Data are expressed as means ± SEM from five independent experiments. *P < 0.01 as compared with vehicle control, **P < 0.005 as compared to vehicle control. (**D**) Changes in pharyngeal pumping rate of aging worms. Pumping rate declines with age, however SA administration retards decline in pumping rate. Data are expressed as means ± SEM from five independent experiments. *P < 0.05 as compared with vehicle control, **P < 0.01 as compared to vehicle control.

The oxidative stress theory of aging postulates that ROS formed by normal metabolic processes play a role in the aging process [37]. The imbalance between pro-oxidants and antioxidants leads to an accumulation of oxidative damage in a variety of macromolecules with age, resulting in a progressive loss in functional cellular processes. Given our observation that the reactive γ-ketoaldehydes termed IsoKs play a critical role in oxidative injury by adducting to and inactivating multiple proteins, we postulated that SA administration would extend natural lifespan by scavenging IsoKs and preventing age-related inactivation of key protein targets. Starting at Day 1 of adulthood, N2 WT *C. elegans* were continuously exposed to increasing concentrations of SA until natural death (Fig. 1A). SA produced a significant dose-dependent increase in median lifespan (Fig. 1B), with 50 μM increasing median lifespan by 18% from 16 days to 19 days, 100 μM increasing lifespan by 32% from 16 days to 21 days (p < 0.05), and 500 μM increasing median lifespan by 56% from 16 days to 25 days (p < 0.01). These data show a significant lifespan extension effect of SA in adult WT *C. elegans*.

We next sought to demonstrate that SA administration would not only prolong natural lifespan in adult worms, but that the longer-lived worms would also exhibit prolonged healthspan – i.e., that they would be pheno-typically more youthful [50]. To quantify this, we chose both a biochemical endpoint (lipofuscin auto-fluorescence) and a behavioral measure (pharyngeal pumping) that change predictably with aging and are associated with healthspan. The accumulation in the nematode intestinal epithelium of autofluorescent lipo-fuscin granules, a heterogeneous mixture of oxidized and crosslinked lipids and proteins and advanced glycation end products, is a known conserved phenomenon observed to increase with age [51,52]. Visualization of lipofuscin granules is often used as an age-related assessment of healthspan. We quantified lipofuscin autofluorescence over time (every 5 days) in N2 adult nematodes (10-20 per colony) in the presence of increasing doses of SA (representative confocal images depicting nematode autofluorescence over age shown in Supplemental Figure 1). SA response profiles were generated from integrating the area-under-the-curve (AUC) of fluorescent intensity as a function of time (Fig. 1C). Treatment with either 100 μM or 500 μM SA showed a significant reduction in age-associated lipofuscin accumulation compared with vehicle control in WT animals (p < 0.01). For a behavioral/functional measurement of health, we quantified pharyngeal pumping rates. Worms ingest bacteria using the pharynx, requiring constant pumping by the pharyngeal bulb [53]. The rate of pharyngeal pumping declines reliably with age and has been attributed to multiple age-related processes [54,55]. We quantified pharyngeal pumping rates in WT N2 worms established on OP50-seeded NGM agar plates with increasing concentrations of SA. The frequency of pharyngeal pumping was measured and recorded every fifth day as the animals aged. Overall, WT N2 worms showed dose-dependent protection against age-associated decline in pharyngeal pumping rate (Fig. 1D) (p < 0.05). Taken together, these data demonstrate that SA not only dose-dependently prolongs lifespan but also healthspan when administered to adult N2 *C. elegans*.

### SA administration deceases formation of IsoK-lysyl-lactam protein adducts

**Figure 2:**
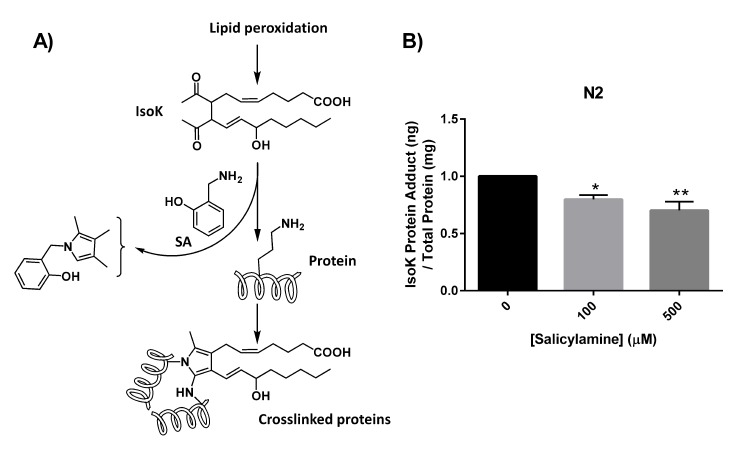
SA administration decreases formation of IsoK-lysyl-lactam protein adducts (**A**) Schematic illustrating lipid peroxidation and formation of IsoKs. Isoks react with ε-amino in lysyl residues of proteins to form stable lactam adducts. Addition of the IsoK scavenger, SA, prevents IsoK adduction. (**B**) IsoK-lysyl-lactam adduct quantification by LC/MS/MS. IsoK-lysyl-lactam adducts were decreased with SA treatment. Data are expressed as means ± SEM from four independent experiments. *P < 0.01 as compared with vehicle control, **P < 0.005 as compared to vehicle control.

The mechanism by which IsoKs inactivate protein targets involves covalent adduction of the epsilon amine group on the side chains of lysine residues within target proteins (Fig. 2A). The initial product of this covalent adduction is a lactam ring structure composed of the lysyl side chain and the isoketal. SA's amine group is much more reactive toward IsoKs and preserves protein function by more rapidly forming an adduct with the IsoK, preventing the adduction of lysyl side chains. If this mechanism is operative *in vivo* when *C. elegans* are treated with SA, a dose-dependent reduction in IsoK-lysyl-lactam adducts should be observed. To test this hypothesis directly, WT N2 worms were treated with vehicle or increasing concentrations of SA until day 15, then collected for IsoK-lysyl-lactam adduct quantify-cation by liquid chromatography tandem mass spectrometry (LC/MS/MS) using a heavy isotope-labeled internal standard for quantification [56]. SA treatment resulted in a significant, dose-dependent reduction in IsoK-lysyl-lactam adduct levels compared with vehicle control in WT animals (p < 0.01) (Fig. 2B).

### SIR-2.1 is a critical protein whose function is preserved by salicylamine

**Figure 3:**
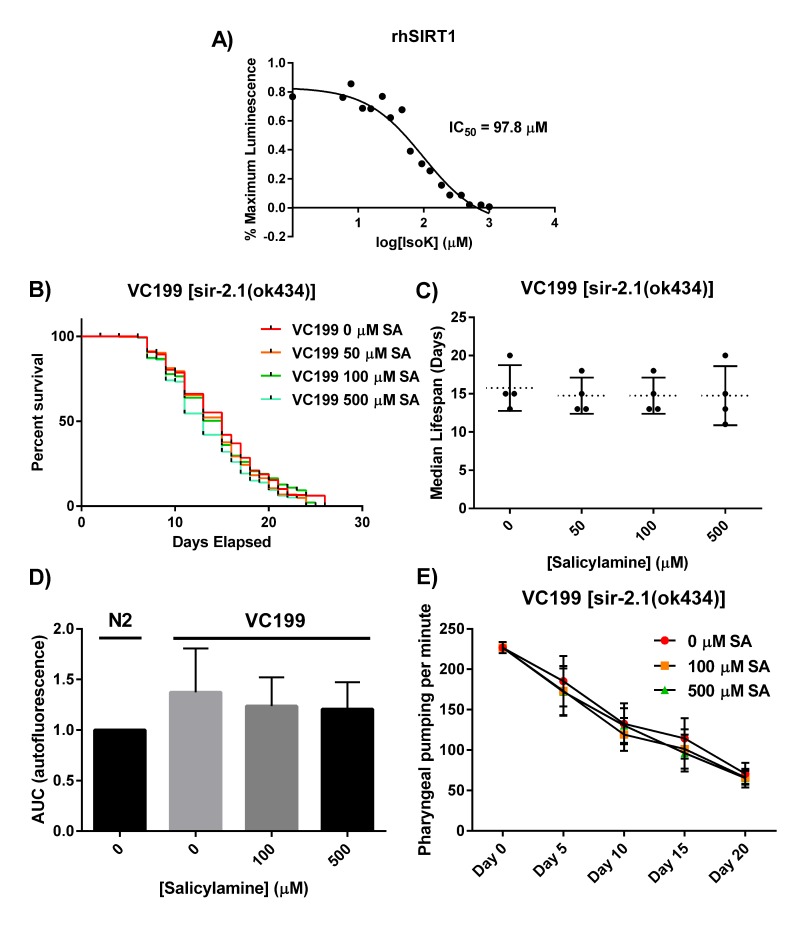
SIR-2.1 is required for SA-mediated lifespan extension (**A**) Synthetically purified IsoKs decrease biochemical activity of rhSIRT1. Recombinant human SIRT1 was incubated with increasing concentrations of IsoK and enzymatic activity was assessed using a luminescence based assay. Concentration-response curves were generated and IC^50^ values were calculated from three independent experiments. (**B**) Kaplan-Meier survival curves depicting effects of SA administration on lifespan of non-functional SIR-2.1 mutant. (**C**) Summary of SA-treated SIR-2.1 mutant median lifespan. SA administration does not affect median lifespan of SIR-2.1 mutants. Data are expressed as means ± SEM from four independent experiments. P = 0.70. (**D**) Changes in lipofuscin autofluorescence accumulation with age. Compared to vehicle control in WT animals, SA response profiles indicate neither dose of SA were able to decrease the accumulation of lipofuscin. Data are expressed as means ± SEM from four independent experiments. P = 0.5. (**E**) Changes in pharyngeal pumping rate in SA-treated SIR-2.1 mutants. Administration of SA failed to preserve pharyngeal pumping rate. Data are expressed as means ± SEM from four independent experiments. P = 0.5.

Though SA treatment should prevent IsoK adduct formation on many different protein targets, and many of these proteins could have some impact on lifespan and healthspan, we hypothesized that there would be specific proteins of particular importance in mediating SA's beneficial effects. Specifically, we hypothesized that SIR-2.1 would be one of these “high value targets” for several reasons. Increased activity of SIR-2.1 and its orthologs have been significantly associated with increased lifespan in multiple studies. Sirtuins are lysine deacetylases that are normally found in membrane-bound and membrane-rich subcellular compartments (e.g., mitochondria), so they are in very close proximity to the membrane lipids that give rise to IsoKs and to the lysyl residues that are their targets. Finally, some sirtuin isoforms have been shown to have greatest deacylase activity toward longer acyl chain lysyl moieties, raising the possibility of an enhanced likelihood of SIR-2.1 being present in close proximity to IsoKs and protein lysyl side chains [57,58]. First, we wanted to directly test the hypothesis that isoketals are chemically capable of inactivating sirtuin proteins, as this would lend plausibility to a direct interaction in vivo and would support the hypothesis that sufficient oxidative stress, rather than activating sirtuins [42,59–62], will actually lead to inhibition. Recombinant human SIRT1 was incubated with increasing con-centrations of synthetically pure IsoK, and enzymatic deacetylase activity was assessed using the luminescence based Sirt-Glo assay (Promega). Purified isoketals dose-dependently inhibited recombinant human sirtuin 1 (Fig. 3A), with an IC^50^ of 97.8 μM.

We next tested the hypothesis that, *in vivo*, SIR-2.1 is a critical protein that is functionally preserved from time-dependent oxidative inactivation when *C. elegans* are treated with increasing doses of SA. We treated a nematode strain carrying a non-functional SIR-2.1 mutation, VC199 [*sir-2.1*(ok434)], with the same concentrations of SA as were used in lifespan experiments with WT nematodes. Starting at day 1 of adulthood, SIR-2.1 mutants were grown on SA-coated OP50-seeded NGM plates. SIR-2.1 mutant worms were transferred to freshly made SA-OP50-NGM agar plates every 2 days and survival was assessed using a platinum wire until all worms died; survival was scored as movement upon slight touch with the platinum wire (Fig. 3B). When SA was administered to the VC199 strain, the effect of extending median lifespan that had been observed in the N2 strain was entirely abolished (Fig. 3C; VC199 median lifespan: 16 days, 0 μM SA; 15 days, 50 μM SA; 15 days, 100 μM SA; 15 days, 500 μM SA; p = 0.70). This suggests that SA-mediated lifespan extension acts in part through preservation of sirtuin activity. In addition, we observed SA-mediated lifespan extension operates outside canonical *C. elegans* longevity pathways, such as the insulin/IGF-1-like signaling pathway (Supplemental Figure 2).

We next wanted to test the hypothesis that SIR-2.1 is required for the healthspan extending effects of SA in addition to the longevity effects. As with the N2 strain, we treated VC199 nematodes with increasing con-centrations of SA and assessed the age-dependent accumulation of autofluorescent lipofuscin granules and decrease in pharyngeal pumping rate. In worms lacking SIR-2.1, none of the doses of SA used were able to decrease the accumulation of lipofuscin (Fig. 3D) (p = 0.50) or to preserve pharyngeal pumping rates (Fig. 3E) (p = 0.50) at day 15. Taken together, these data strongly suggest that SIR-2.1 is a critical target for SA's effects on healthspan as well as lifespan in adult *C. elegans*.

### SIR-2.1 preservation enhances resistance to oxidant stress but does not affect mitochondrial function

**Figure 4:**
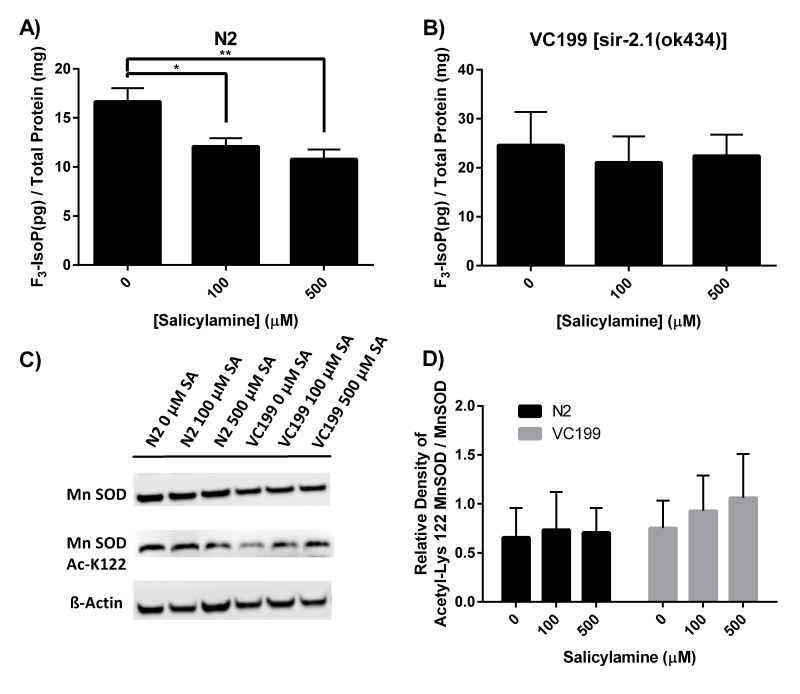
SA treatment dose-dependently decreases biomarkers of oxidant injury in a SIR-2.1-dependent manner (**A**, **B**) Quantification of oxidant damage via F_3_-IsoP measurement. N2 WT and SIR-2.1 mutant animals were given SA from day 1 of adulthood until collection. Lysates were collected at day 15 of adulthood and F_3_-IsoPs were measured by GC/MS. Data are expressed as means ± SEM from four independent experiments. * P < 0.01 as compared with vehicle control, **P < 0.005 as compared to vehicle control. (**C**) Levels of acetyl-Lys 122 MnSOD was measured from N2 WT and SIR-2.1 mutant protein extracts and analyzed by Western blot. (**D**) Quantification of acetyl-Lys 122 MnSOD. Treatment with SA in WT N2 nematodes show a trend toward lower acetyl-Lys 122 MnSOD compared to SIR-2.1 mutant animals. Data are expressed as means ± SEM from four independent experiments. P > 0.05.

Sirtuins represent a regulatory hub for a variety of cellular processes that lie at the heart of molecular aging, including energy metabolism, mitochondrial structural and genomic integrity maintenance, and redox balance. Indeed, one of the major stimuli for activation of sirtuins is oxidative stress. Multiple sirtuin isoforms in multiple different species have been shown to play an important role in cellular defenses against oxidant injury [42,61,62]. The mitochondrial antioxidant manganese superoxide dismutase (MnSOD) has been identified as one of the major protein targets that is deacetylated by sirtuin isoforms, with deacetylation enhancing the enzymatic activity of MnSOD [60]. If SA indeed preserves SIR-2.1 enzymatic function in vivo, we hypothesized that SA treatment would dose-dependently decrease biomarkers of oxidant injury in a SIR-2.1-dependent manner. To quantify oxidant injury, we measured F^3^-isoprostanes (F^3^-IsoPs). F^3^-IsoPs are the products of free radical-mediated peroxidation of eicosapentaenoic acid (EPA), and are known to be a highly sensitive and accurate marker of oxidative damage in *Caenorhabditis elegans* [63–65]. F^3^-IsoPs were collected from WT N2 and from VC199 (SIR-2.1 deficient strain) nematodes grown on OP50-NGM agar plates containing increasing concentrations of SA from Day 1 of adulthood until collection at Day 15. Quantification of F^3^-IsoPs from WT N2 worms (Fig. 4A) exhibited a dose-dependent decrease in F^3^-IsoP levels, with 100 μM SA decreasing F^3^-IsoP production by 29% (p < 0.01) and 500 μM displaying a 44% decrease in F^3^-IsoP levels (p < 0.005). In sharp contrast, SIR-2.1 deficient nematodes showed a slightly higher baseline level of F^3^-IsoPs that did not significantly decrease at any dose of SA tested (Fig. 4B), confirming that SA itself does not act as a direct antioxidant and supporting the hypothesis that SIR-2.1 enzymatic activity is preserved by SA treatment with the predicted positive effect on cellular defense against oxidant injury. To further characterize SA's ability to preserve SIR-2.1 function, we assessed acetylation of MnSOD at Lys 122. At the highest dose of SA (500 μM) on day 15 of adult life, WT N2 nematodes show a trend toward lower acetyl-Lys 122 in MnSOD compared to the VC199 strain treated with the same SA dose (Fig. 4C and 4D), supporting at least a modest positive effect of SA treatment on MnSOD acetylation state and function, mediated by SIR-2.1.

**Figure 5:**
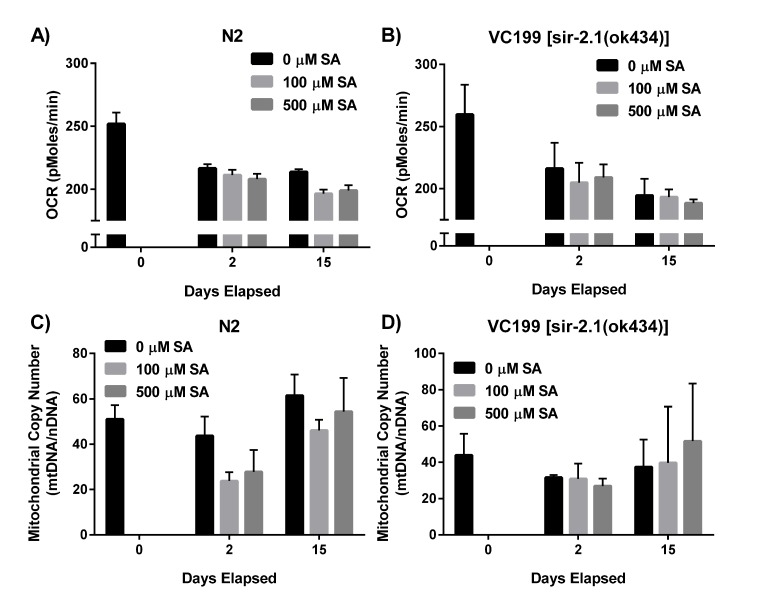
SIR-2.1 preservation does not affect mitochondrial function (**A**, **B**) SA administration does not alter oxygen consumption rate (OCR). OCR of N2 WT and SIR-2.1 mutation in the presence and absence of SA was measured over time via XF Seahorse Biosciences Analyzer™. Data are expressed as means ± SEM from four independent experiments. P = 0.1 and P = 0.3, respectively. (**C**, **D**) SA treatment does not alter mtDNA integrity. Analysis of mtDNA content collected over time from lysates of SA-treated N2 WT and SIR-2.1 mutant animals. Data are expressed as means ± SEM from four independent experiments. P = 0.1 and P = 0.6, respectively.

Mitochondria lie at the center of aging biology, playing crucial roles in energy production, carbon substrate metabolism, apoptosis regulation, and redox balance and signaling [66–68]. Since sirtuins play a major role in regulating mitochondrial function, we next wanted to investigate whether SA was exerting any of its effects via protection of mitochondrial processes. To investigate SA's effects on mitochondrial respiration, we administered SA to WT N2 (Fig. 5A) and non-functional SIR-2.1 mutant worms (Fig. 5B) and measured oxygen consumption rate (OCR) in whole worms over several days. OCR decreased with age in both N2 and VC199 strains, and SA showed no effect on mitochondrial OCR in either strain (p > 0.05). We also examined the effect of SA treatment on mitochondrial DNA (mtDNA) integrity. Using a quantitative polymerase chain reaction (qPCR) assay to measure mtDNA content relative to nuclear DNA, WT N2 (Fig. 5C) and VC199 SIR-2.1 mutants (Fig. 5D) showed no significant difference in mtDNA copy number with age or with SA treatment. Taken together, these data suggest that the SIR-2.1 dependent effects of SA treatment are not mediated through significant changes in mitochondrial function.

### Gene expression analysis reveals *ets-7* as an important effector of salicylamine

**Figure 6:**
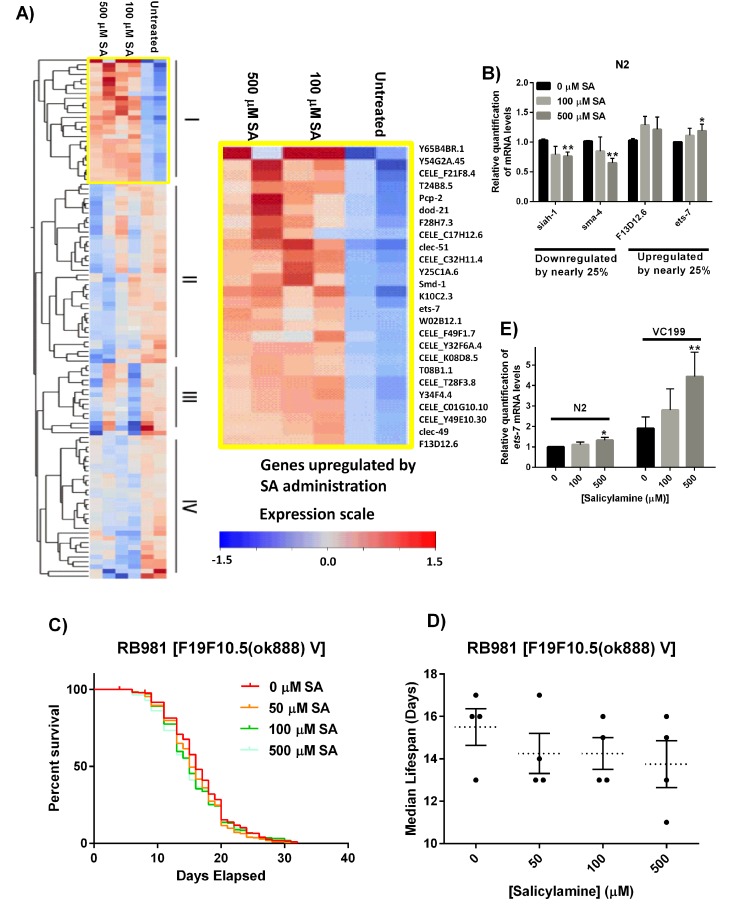
Gene expression analysis reveals *ets-7* as an important effector of SA (**A**) Heat map of genes differentially regulated by treatment in 15 day CE. 109 probe sets had at least a 25% change in expression concordant in both samples. These include 26 genes upregulated by both doses of SA (group I), 38 genes more strongly downregulated by 500 μM SA than 100 μM SA (Group II), 15 genes with variable downregulation (Group III), and 30 genes downregulated regardless of dose of SA (Group IV). (**B**) Real-time RT-PCR validation of microarray results on selected genes. The genes, *siah-1* and *sma-4* showed downregulation by SA in day 15 WT N2 worms, and *F13D12.6* and *ets-7* showed upregulation by SA. Data are expressed as means ± SEM from five independent experiments. *P < 0.05 as compared to vehicle control, **P < 0.01 as compared to vehicle control. (**C**) Kaplan-Meier survival curves for concentration dependency of SA-mediated *ets-7* knock-out mutant lifespan extension. (**D**) Summary of SA-treated *ets-7* knock-out mutant median lifespan. SA administration does not affect median lifespan of *ets-7* knock-out mutants. Data are expressed as means ± SEM from five independent experiments. P = 0.40. (**E**) Real-time RT-PCR quantification of *ets-7* in non-functional SIR-2.1 mutant nematodes treated with increasing doses of SA. Transcriptional levels for ets-7 were increased by 25% in day 15 N2 WT worms by SA administration, and a dose-dependent increase in *ets-7* mRNA levels can be observed in day 15 SIR-2.1 mutants. Data are expressed as means ± SEM from four independent experiments. *P < 0.05 as compared to vehicle control, and **P < 0.01 as compared to vehicle control.

Programmed aging is one of the major theories of aging, wherein this theory implies there is a built-in program in the genome that activates senescence, which leads to death [69]. In addition to the functions discussed above, sirtuins can have powerful regulatory effects on gene expression programs by virtue of their proposed histone deacetylase activity. With supporting evidence that SA's effects are at least in part SIR-2.1 mediated, we sought to better define the role of changes in global gene expression following SA treatment in WT N2 worms. We hypothesized that there would be fairly broad changes in gene expression that would converge on one or more specific pathways. To assess whether SA alters an aging gene transcriptional program, we carried out microarray analysis on adult WT N2 worms exposed to SA (0, 100, 500 μM SA) for 15 days. Gene expression arrays showed that, surprisingly, SA treatment exerted a relatively minor effect on gene expression, with the major variable impacting on gene expression being aging itself. From the microarray, we identified 26 genes upregulated by both 100 and 500 μM SA (Group I), 38 genes more strongly down-regulated by 500 μM SA than 100 μM SA (Group II), 15 genes with variable downregulation (Group III), and 30 genes downregulated by both doses of SA (Group IV) (Fig. 6A). Of the 8,902 probe sets with UniGene identifiers, only 109 probe sets showed at least a 25% change in expression with SA administration at Day 15. Thus, the major effect of SA treatment appears to be at the post-translational level, as would be predicted by SA's proposed mechanism of action.

Nonetheless, there were some significantly changed genes downstream from SA treatment. First, to validate the microarray results, we performed real-time RT-PCR on four genes, *siah-1*, *sma-4*, F13D12.6, and *ets-7* (Fig. 6B). The genes siah-1 and sma-4 showed down-regulation by SA in Day 15 WT N2 worms, and F13D12.6 and *ets-7* showed upregulation by SA. Messenger RNA levels for *siah-1* and *sma-4* were decreased by nearly 25%, and F13D12.6 and *ets-7* message levels were increased by nearly 25% by SA administration (p < 0.05). We did not observe SA effects on *sir-2.1* mRNA levels from the gene expression arrays. Real-time RT-PCR confirmed administration of SA did not appreciably increase or decrease *sir-2.1* transcript levels in aged animals (Supplemental Figure 3), supporting the hypothesis that SA-mediated lifespan extension is acting primarily via preservation of protein biochemistry.

Pathway Analysis using Gene Ontology (GO) with WEB-based Gene SeT AnaLysis Toolkit (WEBGESTALT < http://bioinfo.vanderbilt.edu/webgestalt/>) highlighted the metabolic process, lipid metabolic process, proteolysis pathways among many others as being altered favorably by the administration of SA (Supplemental Figure 4). From the array data confirmed by RT-PCR, we identified the ETS class transcription factor ETS-7 as a protein of interest. ETS factors are known to be involved in regulating lipid metabolism and regulate lifespan in both *Drosophila melanogaster* and *C. elegans* [70,71].

To investigate the role of ETS-7 in regulating lifespan downstream from salicylamine, SA was administered to the *ets-7* gene knock-out strain, RB981 [F19F10.5 (ok888) V]). Similar to the SIR-2.1 deficient VC199 strain, loss of *ets-7* showed no SA-mediated effect on lifespan extension (RB981 median lifespan: 16 days, 0 μM SA; 15 days, 500 μM SA, p > 0.05) (Fig. 6C and 6D). We originally hypothesized that upregulation of *ets-7* depends on SIR-2.1, which could result in enhanced longevity. In order to test this, we carried out real-time RT-PCR quantification of *ets-7* in non-functional SIR-2.1 mutant nematodes treated with increasing doses of SA (Fig. 6E). Messenger RNA levels for *ets-7* were increased by 32% in Day 15 WT N2 worms by 500 μM SA administration (p < 0.05), and similarly, a dose-dependent increase in *ets-7* transcriptional level can be observed in Day 15 non-functional SIR-2.1 mutants, VC199, with the highest dose of SA showing a significant 442% increase in mRNA expression (p < 0.01). Taken together, these findings suggest that *ets-7* upregulation in non-functional SIR-2.1 mutant may be an attempt at compensation for loss of SIR-2.1, which is further enhanced by SA-administration, but is ultimately insufficient in extending lifespan in the absence of SIR-2.1. Our data suggest that *ets-7* is necessary for SA-dependent increase in lifespan, but is insufficient without the presence of functional SIR-2.1.

## DISCUSSION

Several aspects of this study are worth particular attention. First, SA treatment was begun and exhibited its effects in adult worms. This is distinct from some other longevity-extending interventions which rely on genetic manipulation or application of a particular treatment or stressor during a critical developmental period [72–75]. Such interventions are certainly informative but are likely to be of limited direct translation potential. Second, though SA exhibited predictable effects on oxidative stress by way of SIR-2.1 mediated activation of antioxidant enzymes (e.g., MnSOD), the lack of effect on gene expression and on mitochondrial function was striking and informative. This suggests that SA is not working through maintenance or modification of large gene expression programs, nor probably through maintenance of overall nuclear and/or mitochondrial genomic integrity, nor through large effects on mitochondrial function. The lack of any apparent effect on mitochondrial oxygen consumption is perhaps a bit surprising, given the central role of SIR-2.1 in mediating the effects of SA treatment. However, as previously mentioned, SIR-2.1 is known to exert a wide range of pleiotropic effects beyond metabolic regulation [45,49,76–78]. Our data could be consistent with preservation of any number of these other functions ascribed to SIR-2.1, though in these investigations, the major effector mechanism identified downstream from SIR-2.1 was antioxidant defense. Finally, SA treatment does not appear to induce any of the stress/hormesis loops [34,75,79,80] that have been shown to impact upon longevity when activated or inhibited.

The identification of ETS-7 by gene expression array and the subsequent finding that ETS-7 is necessary but not sufficient for SA to exert its effects deserves specific mention. We focused on ETS-7 particularly because there is precedent in the literature for ETS family transcription factors regulating longevity through effects on lipid metabolism [70,71,81]. Given that other lipid metabolic genes were shown to be significantly regulated by SA treatment (Supplemental Tables 1 and 2), we reasoned that ETS-7 may be an important regulatory gene, with the other lipid metabolism genes identified being downstream from ETS-7. Future investigations will focus on the specific gene targets regulated by ETS-7. The finding that ETS-7 is required for SA's effects, but DAF-16 is not (Supplemental Figure 2), also suggests that there are indeed specific protein targets modulated by SA. Furthermore, the observed interactions between SA, SIR-2.1, and ETS-7 strongly suggest that extension of natural lifespan by SA treatment occurs through preservation of the biochemical activities of multiple regulatory proteins, with SIR-2.1 serving as a primary node in the signaling pathway and ETS-7 playing a secondary role. We have certainly not exhaustively investigated all of the possibilities, with other targets (e.g., PCH-2 [82]) existing in the published literature that may be of particular interest. Further delineation of all of the important signaling nodes impacted by SA treatment and elucidation of the signaling hierarchy will be major areas of focus for future investigations.

Our study does have some important limitations. Our data were generated exclusively in *C. elegans*. While a powerful model system for aging research, and though SA treatment has been shown to have substantial beneficial effects in a variety of mammalian models of disease, the findings in the present study will need to be verified in more complex model organisms. A precise dose-effect relationship with SA treatment cannot be accurately determined from the studies we report here. The dose-dependent reduction in IsoK-lactam adducts quantified by mass spectrometry (Figure 2B) confirms that we are in the pharmacologic range, but the precise location on the dose-response curve is not clear. As mentioned above, we also know that there are other protein targets that are likely being preserved with SA treatment, but a full characterization of the proteome-level effects of SA is beyond the scope of the present study.

The translation potential for SA as a clinically useful anti-aging therapy is fairly high. Salicylamine is orally bioavailable in mammals [21]. Long-term administra-tion (approximately one year) in mice via drinking water has shown no evidence of intolerance and no evidence of excess adverse events [22]. This is particularly important when considering an anti-aging intervention, as we would anticipate that SA would need to be administered on an ongoing basis over a long period of time given its mechanism of action. No excess tumor formation was observed in mice with long-term SA treatment [83, 84, Egnatchik RA et al. 2016, unpublished data], an important negative given recent reports regarding negative effects of nonspecific antioxidant therapies with regard to tumor metastasis [85,86]. Finally, the translation of SA into human studies should be able to proceed fairly rapidly, as it is a naturally occurring small molecule found in buckwheat seeds and is currently awaiting Generally Recognized as Safe (GRAS) designation for use as a natural supplement.

## MATERIALS AND METHODS

### *C. elegans* strains and maintenance

*C. elegans* strains were cultured at 20°C on standard nematode growth media (NGM) agar plates seeded with *Escherichia coli* strain NA22. The following strains were used in this work: wild-type *C. elegans* Bristol strain (N2), *sir-2.1(ok434) IV, F19F10.5(ok888) V, and daf-16(mu86)*. Strains were obtained from the Caenorhabditis Genetics Center (University of Minnesota, St. Paul, MN). For generating cultures of 15-day-old (Day 15) adult worms, synchronized late-stage L4s/early young adult worms [87] were transferred to peptone enriched 15 cm plates containing UV-irradiated OP50 *E. coli* and 0.12 mM 5-fluoro-2′-deoxyuridine (FUDR) to inhibit progeny production [88] with or without drug until harvest.

### Salicylamine exposure

Nematodes grown on NGM-agar plates containing 0.5% peptone, were harvested, and eggs were isolated by alkaline hypochlorite with 0.5 N NaOH, 1% hypo-chlorite; 8 min at 23°C. The recovered eggs were rinsed in M9 buffer and placed on fresh agar plates seeded with *E. coli* strain OP50 and maintained at 20°C until late-L4/young adult stage. After the late L4/young adult molt, worms were transferred to peptone enriched 15 cm plates containing 0.12 mM FUDR, OP50 *E. coli*, and varying concentrations of SA. Salicylamine drug plates were made fresh before transfer by spreading SA on top of the agar and plates were allowed to dry. *E*. *coli* strain OP50 was exposed to UV radiation for 30 minutes to kill the bacteria before seeding onto the SA-FUDR NGM agar plates. Worms were exposed to SA throughout its life until harvest by transferring worms to fresh SA-FUDR-OP50 NGM plates every other day.

### Longevity assays

Survival cultures were grown on 60-mm agar plates; after the late-stage L4/young adult molt, approximately 100 adults were transferred onto SA-OP50-seeded NGM plates. Salicylamine drug plates were made fresh before transfer by spreading SA on top of the agar. Plates were allowed to dry before seeding with UV-irradiated OP50 bacteria. Worms were maintained at 20°C and live worms were counted during transfer to freshly made SA-OP50-NGM agar plates every 2-3 days. Survival was scored as movement upon slight touch with the platinum wire. Worms were maintained until death.

### Autofluorescence measurement

Synchronized late L4/early young adult worms were plated on FUDR containing SA-OP50-seeded NGM plates and worms were maintained at 20°C. Every fifth day, 10-15 worms were mounted onto 2% agar pads and anesthetized with 3 mM levamisole in DMSO. Images were taken at 250-ms exposure under a DAPI filter using an epifluorescence microscope (Nikon Eclipse 80i) equipped with a Lambda LS Xenon lamp (Sutter Instrument Company) and Nikon Plan Fluor 20x dry and Nikon Plan Apo 60x 1.3 oil objectives. The fluorescence was calculated using ImageJ software [89].

### Pharyngeal pumping

*C. elegans* pharyngeal pumping rate assays were performed on 60-mm agar plates with bacterial lawns at room temperature. Every fifth day, worms were transferred to fresh bacteria-seeded NGM plates, and incubated at 25°C for 10 min in order to equilibrate feeding rates before measurement. After 10 min incubation, worms were observed under the Zeiss TLB 3.1 microscope with focus on the pharynx. The number of contractions in the terminal bulb of the pharynx was counted for 20 s and then plotted.

### Oxygen consumption analysis

Oxygen consumption rate for whole *C. elegans* was measured using a Seahorse Bioscience XFe96 Analyzer. Worms were harvested from Day 0, 2, and 15 colonies maintained on FUDR containing SA-OP50-seeded NGM plates by washing in M9 medium, followed by floatation on an ice-cold 60% w/v sucrose gradient to segregate clean bacteria-free adult worms from bacterial debris. Worms were seeded at 1,000 worms/well in M9. After 20 min equilibration, a 2-min measurement was performed to obtain basal OCR for all experimental conditions and strains.

### Genome copy number analysis

Relative mitochondrial and nuclear copy number were measured by quantitative, real-time PCR [90]. Primers for NADH dehydrogenase unit 1 (*nd1*) and a 164bp region of the *cox-4* gene were used in determination of mtDNA copy number. The *nd1* forward primer 5′ – AGCGTCATTTATTGGGAAGAAGAC – 3′ and reverse primer 5′ AAGCTTGTGCTAATCCCATAAATGT – 3′. *Cox-4* forward primer 5′ – GCC GAC TGG AAG AAC TTG TC – 3′ and reverse primer 5′ – GCGGAGATCACCTTCCAGTA – 3′. Real-time PCR conditions were 2 min at 50°C, 10 min at 95°C, followed by 40 cycles of 15 s at 95°C, and 60 s at 63°C. Amplified products were detected with SYBR Green (iQ™ SYBR® Green Supermix, Bio-Rad) and fluorescent signal intensities were determined by CFX96 Touch™ Real-Time PCR Detection System (Bio-Rad) by software CFX Manager™ (version 3.1). Crude worm lysate was harvested from Day 0, 2, and 15 stage nematodes grown on FUDR containing SA-OP50-seeded NGM plates and used as template DNA for real-time PCR based determination of mtDNA and nucDNA copy numbers.

### NAD^+^-dependent deacetylation in bioluminescence assay

Relative activity of the NAD**^+^**-dependent histone deacetylase (HDAC) class III enzymes (sirtuins) was measured using the SIRT-Glo™ Assay and Screening System (Promega Corporation, Madison, WI) according to the manufacturer's instructions with minor modifications. This assay uses an acetylated, lumino-genic peptide substrate that can be deacetylated by SIRT activities. Deacetylation of the peptide substrate is measured using a coupled enzymatic system with a protease in the reagent provided and then cleaves the luminogenic peptide to liberate aminoluciferin. Free aminoluciferin can be quantified using the Ultra-Glo™ firefly luciferase reaction to produce a stable, persistent emission of light. Purified recombinant human SIRT1 (R&D Systems, Biotechne) activity was assayed in HEPES-buffered saline (10 mM HEPES, 150 nM NaCl, 2 mM MgCl2) in the presence and absence of 15-E^2^-IsoK. 15-E^2^-IsoK was synthesized by the method of Armanath et al [91]. Luminescence was detected by a microplate reader (FLUOstar Optima microplate reader, BMG Labtechnologies).

### Sample preparation and detection of endogenous F^3^-IsoPs by GC/MS

F**^3^**-Isoprostanes were quantified from SA-treated worms a gas-chromatography-negative ion chemical ionization-mass spectrometry (GC-NICI-MS) approach [92]. Worms maintained on FUDR containing SA-OP50-seeded NGM plates were harvested at Day 15 by washing in M9 medium, followed by floatation on an ice-cold 60% w/v sucrose gradient to segregate clean bacteria-free adult worms from bacterial debris. Clean worms were transferred to Eppendorf tubes and homogenized using the Mini-Beadbeater-24**^®^** (BioSpec, Bartlesville, OK) with zirconium oxide beads (1.0 mm), at 4°C. Homogenates were then hydrolyzed by 15% w/v KOH, containing 57 μM BHT (5% w/v BHT:MeOH) for 30 min at 37°C. Next, samples were centrifuged at max speed to pellet worm debris and supernatant was transferred to a 16-mL polypropylene tubes (Denville Scientific, Inc., Holliston, MA).

Samples were spiked with 248 pg of deuterated internal standards, [**^2^**H**^4^**]-15-F**^2t^**-IsoP, quantified and calibrated by the method of Milne et al. [93] and acidified to pH < 3 with HCl in preparation for further Separation Phase Extraction (SPE). C18 Sep-Pak cartridges (Waters, Milford, MA) were preconditioned with 5 mL of MeOH, followed by 5 mL of pH 3 water and subjected to vacuum to obtain a flow rate of 1 mL/min. Samples were applied to the cartridges and allowed to flow through completely before adding equal volume of pH 3 water and heptane to wash columns before eluting with ethyl acetate: heptane (1:1 v:v). Anhydrous sodium sulfate was then added to each sample to absorb excess water from samples and then applied to silica Sep-Pak cartridges (Waters, Milford, MA) preconditioned with ethyl acetate. Samples were transferred to the silica Sep-Pak columns and allowed to pass through before washing with ethyl acetate, and eluted with ethyl acetate: MeOH (45:55 v:v).

Eluates were dried under nitrogen and F**^3^**-IsoPs and resuspended in MeOH for separation by Thin Layer Chromatography (TLC). The free acid TLC standard, 8-iso-Prostaglandin F**^2α^** methyl ester (8-iso-PGF**^2α^**, Cayman Chemicals, Ann Arbor, MI) and samples were spotted on pre-washed silica TLC plates, placed in a TLC tank containing chloroform: MeOH: Acetic acid (84.5:14.5:1 v:v:v), and allowed to run until reaching solvent front. The free acid TLC standard was visualized by spraying standard plate with phosphomolybdic acid solution, and samples were scraped from the TLC plate in the region of the TLC standard (R**^f^** ~ 0.35). Samples were extracted from silica by resuspension in ethyl acetate: EtOH (1:1 v:v) and dried under nitrogen. All steps from this point followed the F**^3^**-IsoP measurement protocol as described by the method of Nguyen et. al. [63]. Deuterated F**^2^**-IsoP standard was measured at *m/z* 573. F**^3^**-IsoP was measured at *m/z* 567. Endogenous F**^3^**-IsoP levels were quantified by comparing the height of the peak containing the derivatized F**^3^**-IsoP to the height of the deuterated internal standard peak.

Protein concentration of nematode homogenates were determined using the bicinchoninic acid (BCA) protein assay as described by the manufacturer (Pierce Protein Biology, Waltham, MA).

### Quantification of isoketal protein adducts using LC/MS

Worms grown on FUDR containing SA-OP50-seeded NGM plates were collected at Day 15 adult stage by washing in M9 medium, followed by an ice-cold 60% w/v sucrose gradient to segregate clean bacteria-free adult worms from bacterial debris. Clean worms were transferred to Eppendorf tubes and flash-frozen in liquid nitrogen and thawed at 37°C 3x. Samples were homogenized using a handheld homogenizer (Polytron PT 1200E, KINEMATICA AG), in buffer containing antioxidants (100 μM indomethacin, 220 μM butylated hydroxytoluene, and 5 mM triphenylphosphine) and 100 μM pyridoxamine dihydrochloride to prevent artifactural generation of IsoK protein adducts during sample processing. Levels of IsoK-lysyl-lactam adduct was measured as previously described [56].

In brief, IsoK protein adducts are measured after enzymatic proteolysis and separation as IsoK-lysyl-lactam adducts by liquid chromatography tandem mass spectrometry (LC/MS/MS) using a heavy isotope labeled internal standard for quantification. Samples were treated with 15% KOH to hydrolyze esterified isoketals and then subjected to complete proteolytic digestion using pronase protease (*Streptomyces griseus*, Calbiochem, San Diego, CA) and aminopeptidase M (Calbiochem, San Diego, CA), consecutively, to release the IsoK-lysyl-lactam adduct. After digestion, 500 pg of a (^13^C_6_)-IsoK-lysyl-lactam internal standard was added to each sample, followed by partial purification of lysyl adducts by solid-phase extraction (SPE) and further purification by preparative HPLC (2690 Alliance HPLC system, Waters, Milford, MA). Isok-lysyl-lactam adducts were then quantified by selective reaction monitoring LC electrospray tandem mass spectrometry for transition from *m/z* 479 → 84 and *m/z* 487 → 90 for internal standard (ThermoFinnigan Surveyer MS pump coupled to TSQ quantum triple-quadrupole mass spectrometer, Thermo Fischer Scientific, Waltham, MA).

Protein concentration of nematode homogenates were determined using the Thermo Scientific Pierce BCA Protein Assay as described by the manufacturer (Pierce Protein Biology, Waltham, MA).

### Western blot

Day 15 adult worms grown on FUDR containing SA-OP50-seeded NGM plates were harvested in M9 medium, followed by floatation on an ice-cold 60% w/v sucrose gradient to segregate clean bacteria-free adult worms from bacterial debris. Clean worms were transferred to Eppendorf tubes containing radioi-mmunoprecipitation assay (RIPA) buffer with protease inhibitor, trichostatin A, nicotinamide, and phosphatase inhibitors and flash-frozen in liquid nitrogen and thawed at 37°C 3x. Twenty to thirty μg of protein were loaded onto a 10% SDS-PAGE acrylamide gel. Proteins were electroblotted onto nitrocellulose membranes, blocked with 0.1% Tween PBS with 5% nonfat milk and 0.05% sodium azide, and western blots were performed with the primary antibodies anti-MnSOD (ab13533, AbCam, Cambridge, MA), anti-acetyl-lysine 122 MnSOD (a generous gift of D.R. Gius, Northwestern University at Chicago, IL, USA; Epitomics, Inc, Burlingame, CA), anti-acetyl-lysine 68 MnSOD (a generous gift of D.R. Gius, Northwestern University at Chicago, IL, USA;Epitomics, Inc, Burlingame, CA), and anti-β-actin (A5316, Sigma, St. Louis, MO). Proteins were visualized by species-appropriate secondary antibodies labeled with horseradish peroxidase (Santa Cruz Biotechnology, Dallas, TX) and chemiluminescent substrate (Amersham ECL Prime Western Blotting Detection Reagent, GE Healthcare, Pittsburgh, PA). Densitometry was obtained with ImageJ.

### Microarray analyses

Total RNA was isolated via the Trizol method. Worms maintained on FUDR containing SA-OP50-seeded NGM plates were harvested at Day 15 by washing in M9 medium, followed by floatation on an ice-cold 60% w/v sucrose gradient to segregate clean bacteria-free adult worms from bacterial debris. Clean worms were transferred to Eppendorf tubes containing Trizol (Life Technologies) and then snap-frozen in liquid nitrogen and thawed at 37°C 3x. Chloroform was added to each sample, followed by precipitation using isopropanol and washing with 75% ethanol. The supernatant was then transferred to an RNeasy MinElute (Qiagen Inc., Valencia, CA) spin column and all steps from this point followed the RNA purification protocols described in the manufacturer's instructions.

This mixture was then vortexed and transferred to a Shredder Column (Qiagen Inc., Valencia, CA) and centrifuged. Eluate from the Shredder Column was transferred to a Preclear Column contained in the Versagene Kit and all steps following protocols described in the kit manual. After isolation, total RNA was reverse transcribed to double-stranded cDNA, amplified, labeled, and fragmented using the NuGEN Ovulation Biotin Kit (San Carlos, CA). Fragmentation was confirmed using an Agilent Bioanazlyer 2100 (Santa Clara, CA) and fragmented, labeled product was hybridized to an Affymetrix *C. elegans* Gene 1.0 ST GeneChip (Santa Clara, CA) according to the manufacturer's protocols.

Microarray data analysis was performed on arrays normalized by Robust Multi-chip Analysis (RMA). The quality controls on samples and on probe sets were performed stepwise to detect the outlying samples and poor probe sets. The Principal Components Analysis (PCA) score plot and hybridization controls plot were applied for sample detection, with at least one sample with log2(expression)>7. Filtering for high-quality data resulted in 109 genes with at least 25% change in expression, which were defined as salicylamine responsive genes. Independent validation of microarray results was performed by examining changes in mRNA expression using RT-PCR methods as described below.

### TaqMan gene expression assay

Total RNA was isolated via the Trizol method, as described previously. Following isolation, 2 μg total RNA was used for cDNA synthesis using the High Capacity cDNA Reverse Transcription Kit (Life Technologies), per manufacturer's instructions. Quantitative real time PCR (Bio-Rad) was conducted using TaqMan Gene Expression Assay Probes (Life Technologies) for each gene. Amplified products were normalized to housekeeping gene, *ama-1* (RNA polymerase II) after determining fold difference using the comparative 2-^ΔΔCt^ method [94]. The following probes were used: *ama-1* (Assay ID: Ce2462269_m1), *ets-7* (Ce02477624_g1), F13D12.6 (Ce02439540_m1), *siah-1* (Assay ID: Ce02462269_m1), and *sma-4* (Assay ID: Ce202447346_g1).

### Statistics

All statistical analyses were performed using GraphPad Prism 6 (GraphPad Software, Inc.). Concentration response curves were generated using a sigmoidal dose-response model with a top constraint at 100%. Statistical significance of the lifespan experiments was assessed using Mantel-Cox log-rank test, a nonparametric measure that assesses differences in entire survival curves. Comparisons between two groups were performed using a two-tailed Student's t-test assuming equal variances. Multiple group comparisons at different time points was done using two-way ANOVA with repeated measures, followed by Bonferroni's multiple comparison post-hoc tests. Values of P < 0.05 were considered statistically significant.

## SUPPLEMENTARY DATA FIGURES AND TABLES


