# Supplementary material for: Scavengers of reactive γ-ketoaldehydes extend Caenorhabditis elegans lifespan and healthspan through protein-level interactions with SIR-2.1 and ETS-7
Source: Aging (Albany NY). 2016 Aug 9;8(8):1759–77. doi: 10.18632/aging.101011 (PMC5032694; doi:10.18632/aging.101011)
Supplement: Supplementary file 1 [file aging-08-1759-s001.pdf]

## SUPPLEMENTAL DATA

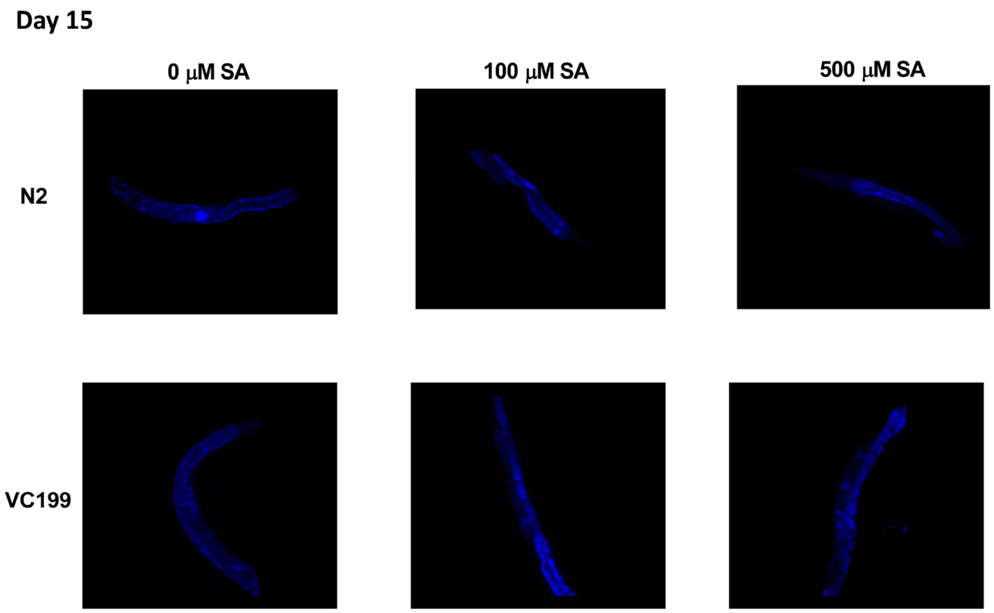


**Figure S1. Change in lipofuscin autofluorescence with age.** Representative confocal images are shown from four experiments. Synchronized late L4/early young adult worms were plated on FUDR containing SA-OP50-seeded NGM plates and worms were maintained at 20°C. Every fifth day, 10-15 worms were mounted onto 2% agar pads and anesthetized with 3 mM levamisole in DMSO. Representative confocal images of each treatment condition were captured through Plan-Aprochromat 20x objective on an LSM510 confocal microscope (Carl Zeiss MicroImaging, Inc) scanning every 200 nm for XZ sections. Images were processed with the Zeiss LSM Image Browser. Figure S1 relates to manuscript figure 1C and 3D.

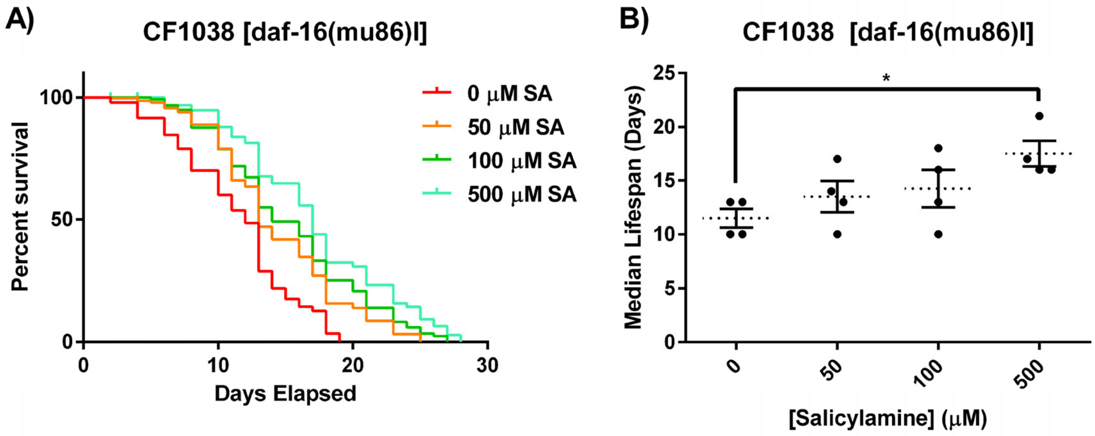


**Figure S2. SA extends the lifespan of *daf-16* gene knockout mutant strain.** (**A**) Kaplan-Meier survival curves depicting effects of SA administration on *daf-16* gene knockout mutant strain. Starting at day 1 of adulthood, animals were transferred to OP50-seeded NGM-SA plates every 2 days. Survival was assessed every 2 days until all the worms died. (**B**) Summary of SA treated *daf-16* knockout mutant median lifespans. SA increased maximum and median lifespan in *daf-16* knockout worms. Data are expressed as means ± SEM from four independent experiments. *P < 0.01 as compared to vehicle control. Figure S2 relates to manuscript figure 3B.

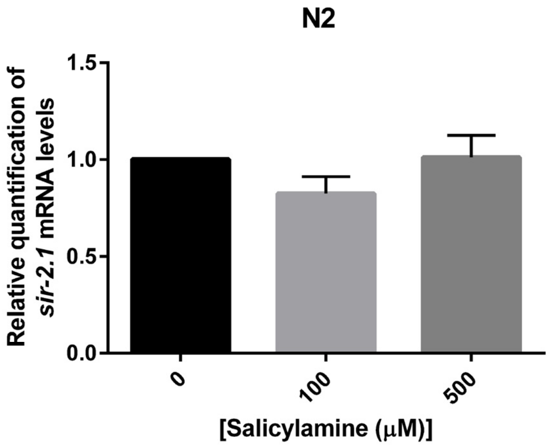


**Figure S3. SA does not attenuate *sir-2.1* mRNA levels.** Real-time RT-PCR quantification of *sir-2.1* in wild-type N2 nematodes treated with increasing doses of SA. Data are expressed as means ± SEM from five independent experiments. P = 0.08 and P = 0.2, respectively.

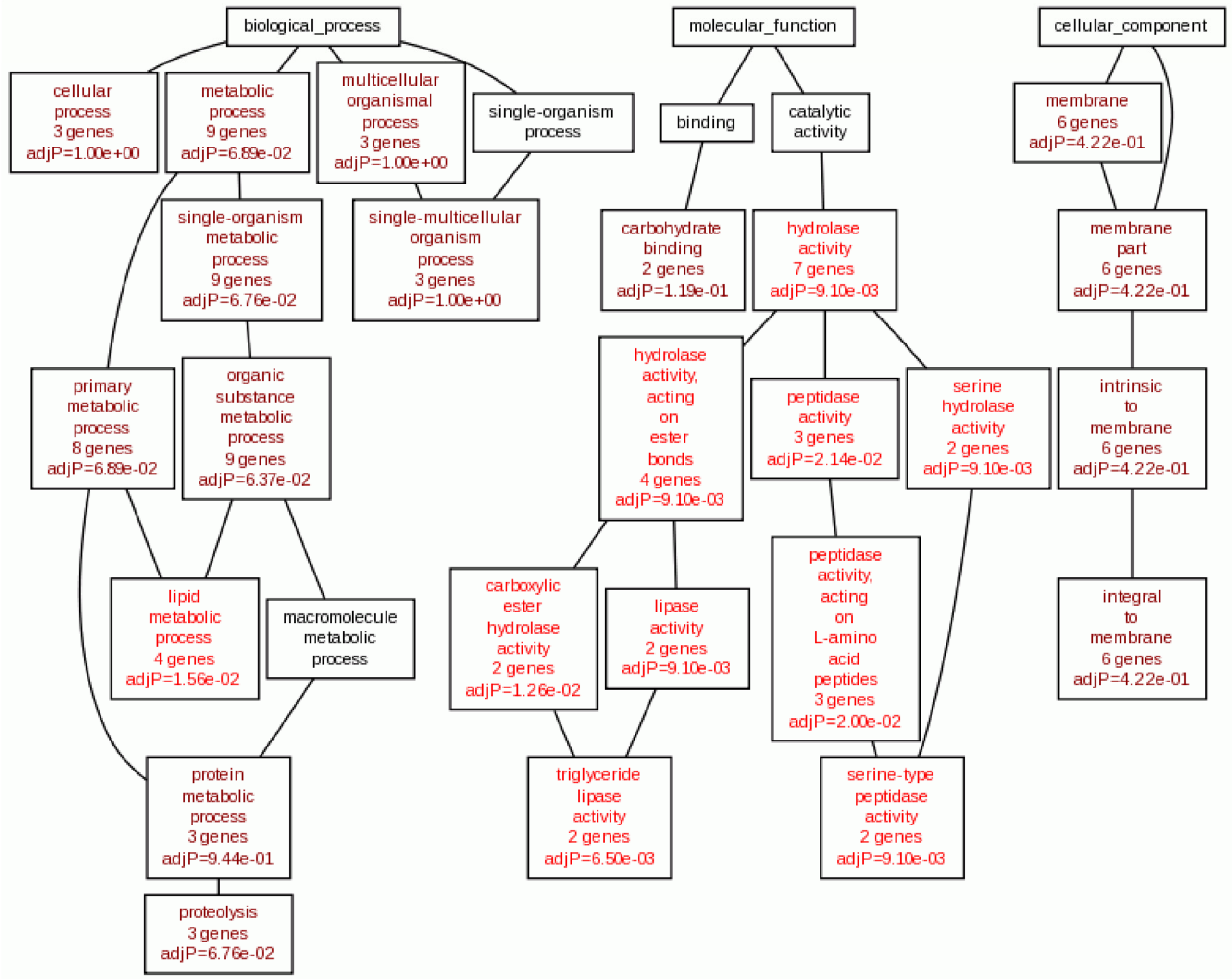


**Figure S4. Gene Ontology enrichment via WEBGESTALT.** Pathway analysis of SA-mediated genomic changes in day 15 N2 WT worms. To further explore the genomic effects of SA administration on N2 WT worms, Gene Ontology (GO) enrichment was performed using WebGestalt, an approach which incorporates information from different public resources and provides graphical depiction of large gene sets from functional genomic, proteomic, and large-scale genetic studies. Biological relationships among Directed acyclic graphs (DAG) were generated using GOView, a web-based application to allow users to visualize and compare multiple provided GO term lists to identify common and specific biological themes. DAG of Group I genes upregulated by SA administration. Chart highlights the metabolic process, lipid metabolic process, and proteolysis pathways among many others as being altered favorably by SA administration.

**Supplemental Table 1. Lipid metabolism genes Identified by GO/WebGestalt analysis.**

| **Lipid Metabolic Process** | | | **ID: GO: 0006629** |
|---|---|---|---|
| **Gene Symbol** | **Gene Name** | **EntrezGene** | **Ensembl** |
| Y65B4BR.1 | Protein Y65B4BR.1 | 190488 | CELE_Y65B4BR.1 |
| W02B12.1 | Protein W02B12.1 | 174746 | CELE_ W02B12.1 |
| F28H7.3 | Protein F28H7.3 | 179490 | CELE_ F28H7.3 |
| Y54G2A.45 | Protein Y54G2A.45 | 3896802 | CELE_ Y54G2A.45 |

List of lipid metabolism genes identified by Gene Ontology/WebGestalt analysis that are significantly upregulated by salicylamine treatment. This is the subset of genes most likely to represent downstream targets of *ets-7*.

**Supplemental Table 2. Metabolic process genes Identified by GO/WebGestalt analysis.**

| **Metabolic Process** | | | **ID: GO: 0008152** |
|---|---|---|---|
| **Gene Symbol** | **Gene Name** | **EntrezGene** | **Ensembl** |
| Y65B4BR.1 | Protein Y65B4BR.1 | 190488 | CELE_Y65B4BR.1 |
| *pcp-2* | Protein PCP-2 | 177741 | CELE_F23B2.12 |
| W02B12.1 | Protein W02B12.1 | 174746 | CELE_ W02B12.1 |
| *ets-7* | Protein ETS-7 | 184687 | CELE_F19F10.5 |
| Y54G2A.45 | Protein Y54G2A.45 | 3896802 | CELE_ Y54G2A.45 |
| *smd-1* | Protein SMD-1 | 173269 | CELE_F47G4.7 |
| F13D12.6 | Protein F13D12.6 | 174802 | CELE_F13D12.6 |
| F28H7.3 | Protein F28H7.3 | 179490 | CELE_ F28H7.3 |
| K10C2.3 | Protein K10C2.3 | 180917 | CELE_K10C2.3 |

The larger list of genes exhibiting significant changes with salicylamine treatment, and reorganized as representing metabolic processes more broadly by GO/WebGestalt. Notably, this list includes all of the genes in Supplemental T1 and captures *ets-7* itself.
